# Myocardial infarction with non-obstructive coronary arteries in a young seropositive woman with human immunodeficiency virus: a case report and review of the literature

**DOI:** 10.1186/s13256-024-04776-w

**Published:** 2024-09-13

**Authors:** Meriem Boumaaz, Raid Faraj, Ahmed Reggad, Zouhair Lakhal, Iliyasse Asfalou

**Affiliations:** 1 Department of Cardiology, Mohammed V Military Hospital, Mohammed V University, Rabat, Morocco; 2 Department of Virology, Mohammed V Military Hospital, Mohammed V University, Rabat, Morocco

**Keywords:** Myocardial infarction, HIV infection, Antiretroviral therapy, Coronary artery disease, Case report

## Abstract

**Background:**

Elevated susceptibility to acute myocardial infarction and various cardiovascular diseases has been observed in individuals infected with the human immunodeficiency virus compared with the uninfected population, as demonstrated in numerous studies. The precise mechanism by which human immunodeficiency virus infection heightens the risk of acute myocardial infarction remains elusive. The manifestation of acute coronary syndrome in young patients with human immunodeficiency virus may deviate from the typical, displaying distinct pathophysiological and clinical characteristics. The occurrence of myocardial infarction with non-obstructive coronary arteries in young patients with human immunodeficiency virus poses diagnostic and treatment challenges.

**Case presentation:**

We present the case of a 46-year-old African woman with no traditional atherosclerotic risk factors. She was diagnosed with human immunodeficiency virus-1 infection 2 years prior to her current admission for chest pain. Her troponin levels were elevated, suggestive of acute coronary syndrome. Although coronary angiography ruled out coronary artery stenosis, it revealed mild myocardial bridging in the left anterior descending artery. Cardiac magnetic resonance imaging confirmed myocardial infarction, indicating a myocardial infarction with non-obstructive coronary arteries with an apical thrombus in the left ventricle. Following medical treatment, the patient experienced resolution of chest pain and improvement in ST-segment elevation.

**Conclusions:**

In young female patients without traditional risk factors, human immunodeficiency virus infection is a possible etiological factor for myocardial infarction with non-obstructive coronary arteries. The likely pathophysiological pathway is superficial endothelial cell denudation as a result of chronic inflammation and immune activation.

**Supplementary Information:**

The online version contains supplementary material available at 10.1186/s13256-024-04776-w.

## Background

As a result of the effectiveness of antiretroviral therapy (ART), the management of human immunodeficiency virus (HIV) has significantly improved, transforming it into a largely controllable condition. However, this advancement has led to a rising prevalence of chronic, non-communicable diseases among those living with HIV. Among these, individuals with HIV face elevated risks of coronary artery disease (CAD) due to various potential factors. Understanding the link between HIV infection, myocardial bridging (MB), and myocardial infarction with non-obstructive coronary arteries (MINOCA) remains limited, yet it likely involves several mechanisms. Chronic inflammation and endothelial dysfunction triggered by HIV and may render individuals more susceptible to microvascular dysfunction and thrombosis and may exacerbate the pathophysiological mechanisms of MB, thereby contributing to MINOCA. Moreover, HIV-related prothrombotic conditions, immune system dysregulation, and endothelial damage may exacerbate myocardial ischemia even in the absence of significant coronary artery obstruction [1].

This case report highlights a rare instance of myocardial infarction with MINOCA in a young woman who is HIV-positive. It underscores the diagnostic and therapeutic challenges posed by the interplay between HIV infection, MB, and MINOCA, offering new insights into myocardial ischemia in the absence of traditional cardiovascular risk factors.

## Case presentation

A 46-year-old Moroccan women, without traditional atherosclerotic risk factors, presented to virology department with complaints of fever and productive cough. Her dental history was unremarkable, with no major dental issues reported. The patient had no history of pregnancies and no history of miscarriages or other gynecological conditions. The patient does not smoke or consume alcohol. She lives in a low-income urban area. There was no family history of coronary artery disease or other cardiovascular conditions. The patient is a homemaker with no occupational exposures or stressors. Her fever was low grade and spiked in the evenings, reaching a maximum of 38 °C. A total of 2 years before current admission, she was diagnosed with HIV-1 infection, and had Atenef (efavirenz 600 mg/emtricitabine 200 mg/tenofovir disoproxil fumarate 300 mg) as anti-retroviral agent treatment. On examination, she was a thin, lean young female. Neurological examination revealed that the patient was fully alert and oriented, with no signs of focal neurological deficits. Cranial nerve examination was normal, with intact motor and sensory functions. Her blood pressure was 143/88 mm Hg, her pulse was 105 beats/min, her respiratory rate was 32 breaths/min, and her weight was 45 kg. On auscultation, crackling rales in the right lung were heard, cardiac auscultation was normal with no added sounds, and oxygen saturation was at 90% on room air.

**Fig. 1:**
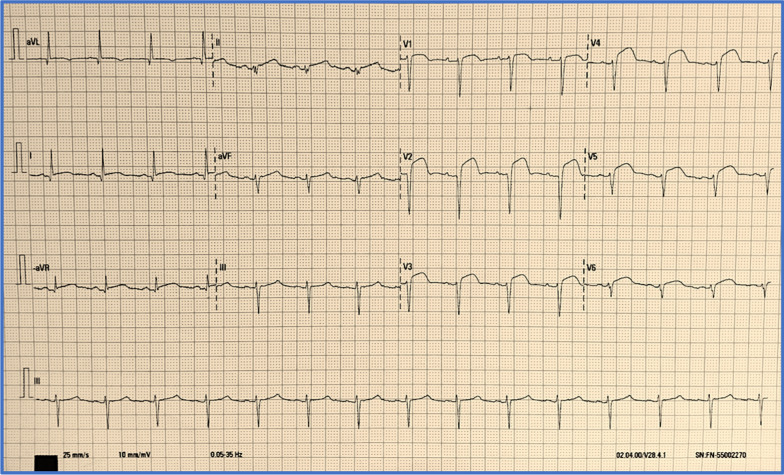
ECG showed a regular sinus rhythm with an elevation of ST segment on the leads from V1 to V6 and D1-aVL

Her chest x-ray showed bilateral perihilar infiltrates, and the diagnosis of pneumocystosis was made on the basis of the presence of *Pneumocystis* *jirovecii* on bronchoscopy sample. Therefore, the patient had a treatment with a high dose of co-trimoxazole. A total of 7 days later, during her hospitalization, she felt an intense and prolonged thoracic pain. Electrocardiogram (ECG) showed an elevation of the ST segment on the leads from V1 to V6 and D1-aVL (Fig. 1) with troponin levels at 14,000 ng/L (normal range: less than 39 ng/L).

**Fig. 2:**
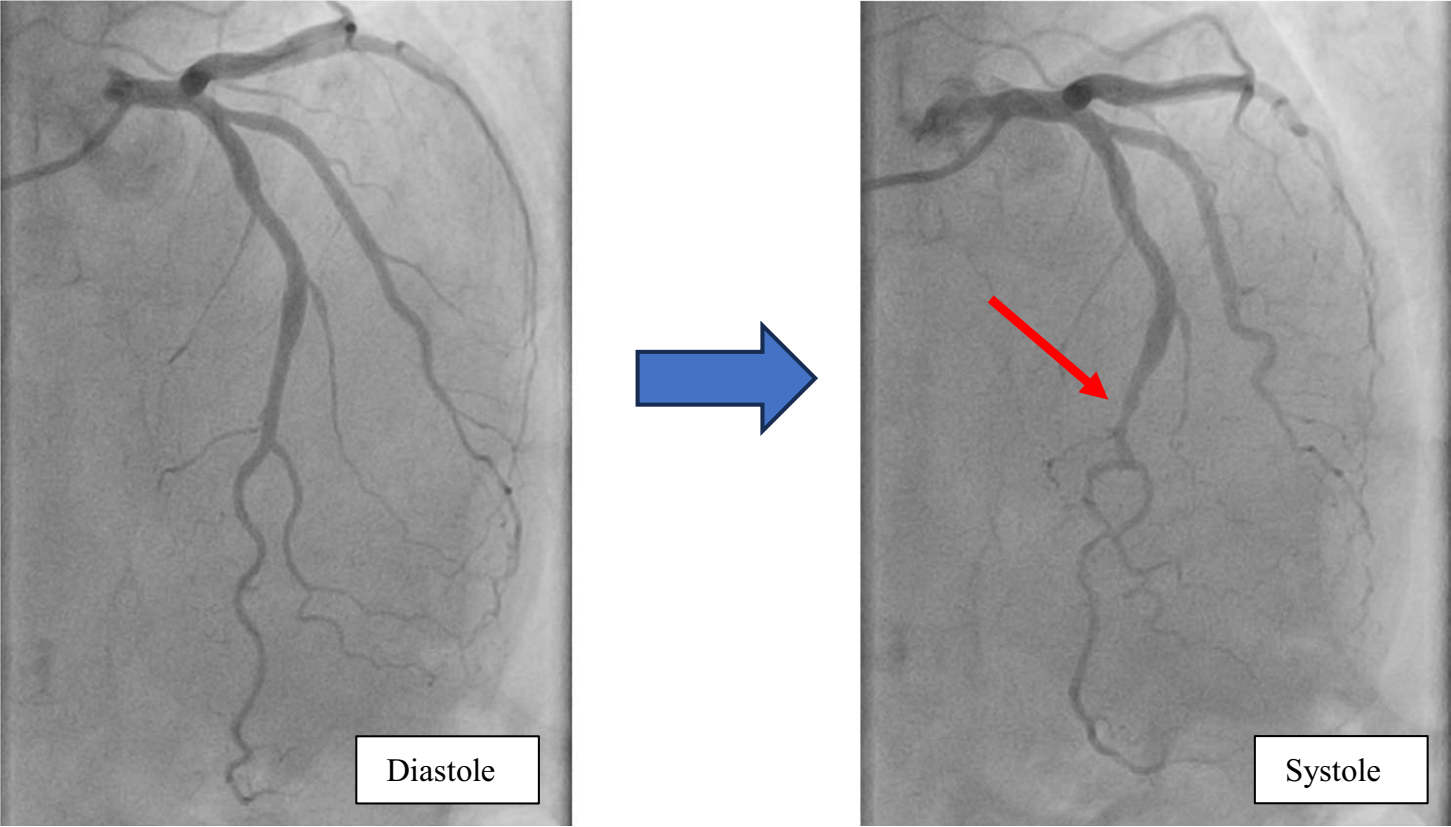
Coronary angiography revealing a mild myocardial bridging in LAD in systole (red arrow)

Transthoracic echocardiography (TTE) noted regional wall motion abnormalities; the apex of left ventricle was akinetic as well as the apical segments of all other walls. Ejection fraction (EF) was at 45%. Emergent coronary angiography (CA) was performed and ruled out coronary artery stenosis but revealed mild MB in left anterior descending (LAD) artery (Fig. 2, Videos 1–2).

**Fig. 3:**
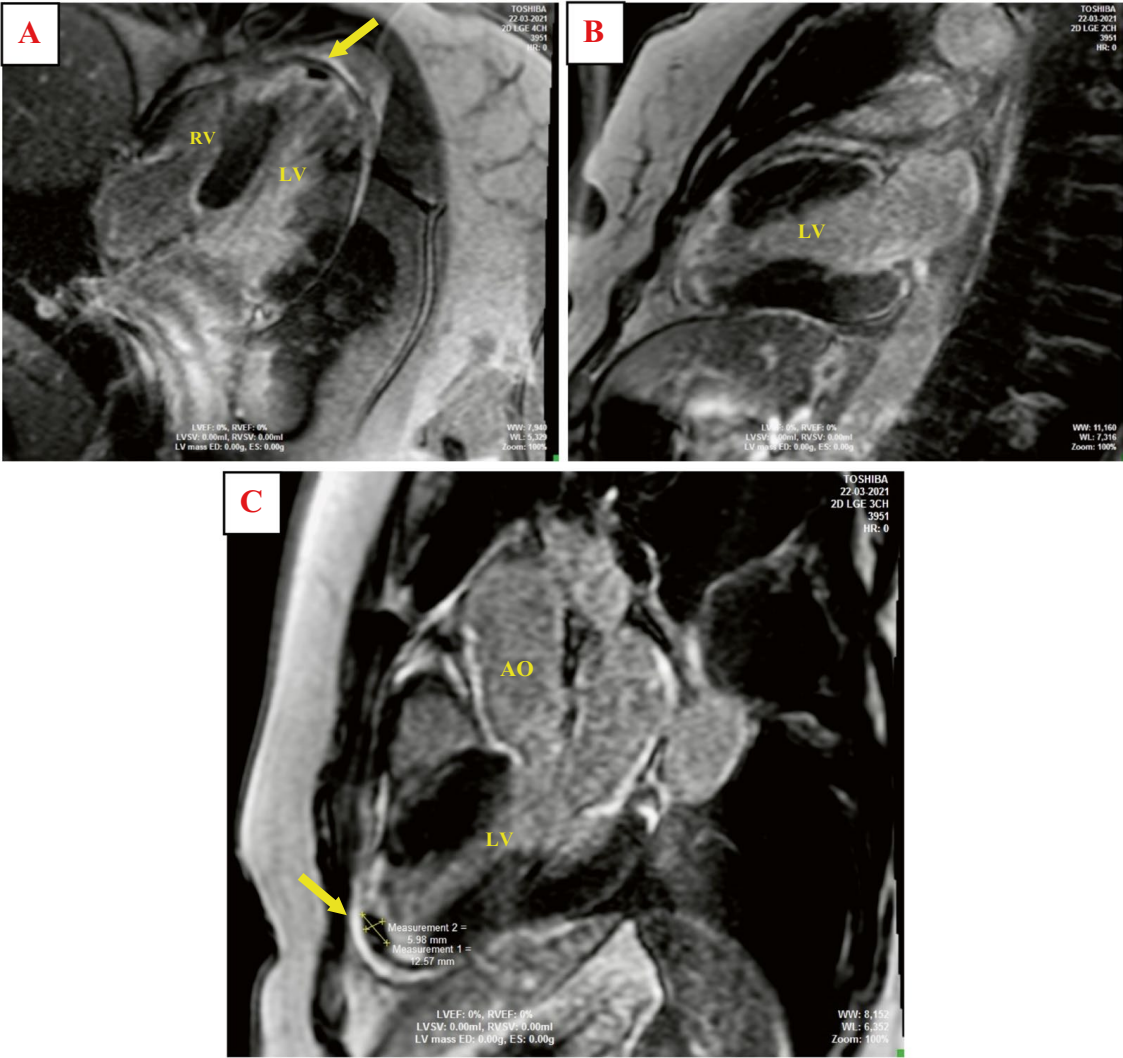
Cardiac magnetic resonance imaging (MRI) sequences of late enhancement in a **A** four-chamber left view and **B** two-chamber left view showing enhancement of the apex and all apical segments of left ventricle walls. **C** MRI sequences of late enhancement in a three-chamber view showing the absence of late gadolinium enhancement of an apical left ventricle mass attesting the diagnosis of intracardiac thrombus measuring 12 mm × 6 mm (yellow arrow); *RV* right ventricle, *LV* left ventricle, *AO* aorta

Cardiac magnetic resonance (CMR) confirmed abnormalities in contractile function and identified an apical thrombus of the left ventricle measuring 12 mm × 6 mm with transmural late gadolinium enhancement (LGE) at the apex and all apical segments of left ventricle walls, suggestive of an ischemic pattern. The right ventricle (RV) was without abnormalities (Fig. 3).

The complete blood count (CBC) indicated mild anemia (hemoglobin 11.2 g/dL), with a low-normal white blood cell count (4500 cells/µL) and normal platelets. Liver function tests showed mildly elevated transaminases (ALT 55 U/L, AST 62 U/L) but normal alkaline phosphatase and bilirubin. Renal function and electrolytes were normal. Inflammatory marker was elevated (CRP 45 mg/L), likely due to pneumocystosis and HIV. Serology showed an HIV viral load of 1200 copies/mL and a CD4 count of 250 cells/µL, indicating controlled infection under ART.

The patient was treated with bisoprolol (administered orally at a dose of 5 mg once daily), ramipril (administered orally at a dose of 5 mg once daily) and acenocoumarol at an initial dose of 2 mg daily, with adjustments made on the basis of regular monitoring of the international normalized ratio (INR) to maintain a therapeutic range between 2.0 and 3.0 and then discharged home. ART was discontinued 1 month before introducing it again. After 2 years of follow-up, no symptomatology recurrence has been noted.

## Discussion

While MINOCA is a known entity, its occurrence in a young patient who is HIV-positive with these specific characteristics adds a novel perspective to the existing literature. This case underscores the importance of considering alternative etiologies, such as microvascular dysfunction and chronic inflammation, in the diagnosis and management of MINOCA, particularly in patients with HIV, where the pathophysiology may differ from the general population.

MINOCA presents as a unique clinical syndrome where myocardial infarction (MI) is evident, yet angiography reveals normal or nearly normal coronary arteries (with stenosis severity ≤ 50%), excluding obvious non-coronary causes of MI such as severe hemorrhage or respiratory failure [2]. Among those experiencing acute MI, MINOCA is detected in 1–15% of cases [3]. It is worth noting that this condition disproportionately affects females and could potentially make up up to half of all MIs in women under 55 years old, and it tends to have a lower presence of traditional cardiovascular risk factors, which is similar to our case.

The pathophysiology of MINOCA encompasses multiple mechanisms, including plaque erosion, coronary artery spasm, microvascular dysfunction, and thrombosis [2]. In HIV infection, chronic inflammation, immune dysregulation, endothelial dysfunction, and hypercoagulability play roles in cardiovascular complications [4]. These overlapping mechanisms imply a potential association between HIV and MINOCA.

Numerous studies have indicated an elevated risk of clinical CAD in persons living with HIV (PLWH). In general, PLWH are estimated to face a 1.5–2-fold higher risk of CAD compared with individuals without HIV (HIV−) [1]. Consequently, HIV infection may be deemed as significant as certain traditional CAD risk factors, such as diabetes and smoking, potentially contributing to improved risk stratification and decision-making in clinical practice. The process of HIV-associated acute coronary syndrome (ACS) appears to lean toward favoring plaque rupture rather than erosion. Nevertheless, a more comprehensive understanding of plaque erosion has unveiled potential pathways that could be significantly involved in chronic HIV infection. In our scenario, the absence of endovascular imaging poses a limitation, as it could have revealed erosion of a vulnerable plaque as a possible cause of MINOCA. MB is a congenital abnormality in which a segment of an epicardial coronary artery, most often the left anterior descending artery, deviates from its usual epicardial path and travels through the myocardium instead. In patients MINOCA, the prevalence of MB can be as high as 20–40% [5]. Recent studies have shown that MB, either alone or combined with other contributing mechanisms, is a frequent but often overlooked cause of MINOCA [6]. In our case, the presence of MB in the context of HIV may exacerbate endothelial dysfunction, as the prevalence of epicardial or microvascular coronary vasospasm is high among patients with both MB and HIV.

The in-depth examination of coronary microvascular structure and function has not received the same level of attention as CAD in general, particularly among individuals living with HIV. The coronary microvasculature, which governs coronary vascular resistance and thus significantly influences coronary blood flow, remains relatively understudied. While the “no-reflow” phenomenon, indicating microvascular dysfunction, is a recognized issue where myocardial reperfusion fails despite epicardial artery recanalization, there is also mounting evidence pointing to microvascular dysfunction’s role in precipitating ACS, even without obstructive epicardial CAD, even among those effectively treated with ART [6], as observed in our case. However, despite the accumulating evidence, the precise underlying mechanisms remain elusive. In a study by Arjun Sinha et al. [7], a comprehensive investigation into the association between various biomarkers and microvascular dysfunction was conducted among individuals who areHIV-positive, including those who were treated and had achieved viral suppression. Interestingly, the research findings revealed significant associations between CD8+PD1+ cells, tumor necrosis factor-alpha, and microvascular dysfunction across all subjects who were HIV-positive, irrespective of treatment status. Moreover, biomarkers such as D-dimer, high-sensitivity C-reactive protein, sCD-14, and interleukin-6 were also found to be linked with microvascular dysfunction in the HIV-positive cohort.

It is notable that women constitute a quarter of HIV cases. Women with HIV experience higher rates and increased risk of cardiovascular disease (CVD) events compared with uninfected women, with a hazard ratio (HR) of 2.8 [95% confidence interval (CI) 1.7, 4.6; *P* < 0.001] [8]. This elevated risk persists even after adjusting for demographics, risk factors, comorbidities, and substance abuse. Additionally, the use of antiretroviral drugs adds further burden [9]. Furthermore, studies indicate that coronary vasospasm, significantly driven by local and systemic inflammation, manifests more prominently in women experiencing myocardial infarction compared with men [10]. This suggests a potential exacerbation of these pathological mechanisms among women who are HIV-positive due to the underlying inflammatory conditions associated with chronic HIV infection. Additionally, HIV infection may heighten the risk of coronary artery disease in women through alternative pathways, such as early or premature menopause resulting from coinfections and immune system dysfunction [10].

## Conclusion

MINOCA presents a unique clinical entity with diverse pathophysiological mechanisms, potentially linked to HIV infection. Further understanding of these mechanisms is crucial for improving risk stratification and management strategies, especially considering the rising prevalence of cardiovascular complications in individuals living with HIV.

## Supplementary Information

Supplementary file 1.

Supplementary file 2.

## Data Availability

Not applicable.
